# Analysis of risk factors for esophagojejunal anastomotic leakage after total gastrectomy based on Bayesian network model

**DOI:** 10.3389/fmed.2025.1632214

**Published:** 2025-08-05

**Authors:** Yun-Feng Wang, Zi-Qi Guo, Jing-Xiang Han, Lin-Na Gao, Yu-Ming Liu, Kai Jia, Hao Chen, Tian Yao, He Huang

**Affiliations:** ^1^The First Clinical Medical College, Shanxi Medical University, Taiyuan, Shanxi, China; ^2^Department of Gastrointestinal Surgery, First Hospital of Shanxi Medical University, Taiyuan, Shanxi, China; ^3^Department of Nutrition and Food Hygiene, School of Public Health, Shanxi Medical University, Taiyuan, Shanxi, China

**Keywords:** gastric cancer, esophagojejunal anastomotic leakage, type of anastomosis, prediction model, Bayesian network model

## Abstract

**Objectives:**

This research aims to develop a nomogram for predicting esophagojejunal anastomotic leakage (EJAL) after total gastrectomy and analyze the relationship between individual risk factors through the Bayesian network model.

**Materials and methods:**

The research enrolled 238 patients who underwent total gastrectomy and esophagojejunal Roux-en-Y anastomosis for gastric cancer between January 2017 and June 2022 in the Department of Gastrointestinal Surgery of the First Hospital of Shanxi Medical University and retrospectively collected clinical data of the patients. Multivariable logistic regression was used to explore the risk factors of EJAL and a nomogram based on the results was constructed. The predictive ability of the model was assessed by receiver operating characteristic (ROC) curve and calibration curve. In addition, the clinical benefit was indicated by decision curve analysis (DCA). Ultimately, a Bayesian network model was developed to analyze the interrelationship between the risk factors.

**Results:**

Esophagojejunal anastomotic leakage occurred in 13 of 238 patients (5.4%). End-to-side anastomosis, diabetes mellitus (DM), preoperative albumin (ALB) ≤ 33.6 g/L, drinking history and systemic inflammation response index (SIRI) > 1.18 were identified as independent risk factors for EJAL based on multivariable logistic regression. A nomogram containing the aforementioned factors was constructed, with an area under the receiver operating characteristic curve (AUROC) of 0.880. Likewise, the model showed good predictive ability and clinical application in the calibration curve and DCA. Ultimately, the Bayesian network model demonstrates that type of anastomosis (ToA), DM, and ALB were directly associated with EJAL development, while gender, age, drinking history, smoking history, hypertension, and SIRI were conditionally dependent on EJAL given the presence of mediator variables.

**Conclusion:**

Surgeons should be alert to the occurrence of EJAL, especially in patients with end-to-side anastomosis, DM, drinking history, preoperative lower ALB, and higher SIRI. Also, males, advanced age, smoking history, and hypertension can affect the development of EJAL.

## 1 Introduction

Gastric cancer (GC) is a malignant tumor that seriously threatens human health, and most patients are already at advanced stages by the time they are detected due to the early mild symptoms and low prevalence of regular screening (1). According to Global Cancer Statistics 2022, there were almost 0.97 million new cases of gastric cancer and 0.66 million deaths globally in 2022, with new cases and deaths ranking fifth (2). Radical surgery remains the mainstay of treatment for gastric cancer. For patients who meet the criteria, total gastrectomy with D1/D2 lymph node dissection is the accepted procedure (3).

Several methods of gastrointestinal (GI) reconstruction after total gastrectomy have been proposed, esophagojejunal Roux-en-Y anastomosis is currently the most commonly used form due to its simplicity and effectiveness in preventing bile reflux (4, 5). In spite of advances in surgical skills and perioperative management, the occurrence of esophagojejunal anastomotic leakage (EJAL) remains inevitable. EJAL is still one of the most prevailing and serious complications after performing a total gastrectomy. The incidence of EJAL has been reported to be ranging from 2.1% to 14.6% (6, 7). Patients who develop an EJAL after surgery have to suffer more anguish and even death (8). According to previous research, EJAL has a fatality rate of up to 50% and is one of the leading causes of postoperative mortality (7, 9). Therefore, timely prediction and intervention are crucial.

End-to-side anastomosis produces superior postoperative outcomes (including anastomotic leakage, postoperative ileus, etc.) compared to side-to-side anastomosis in a cohort control research of right colectomy (10). However, the relationship between the type of esophagojejunal anastomosis and EJAL has not been articulated. While multiple risk factors including advanced age, male gender, diabetes, alcohol history, hypoalbuminemia, elevated SIRI, preoperative anemia, major blood loss, and prolonged operation time have been associated with EJAL in prior studies, the interdependencies among these variables remain poorly characterized due to limitations in conventional statistical methods (9, 11–15). Bayesian network (BN) can intuitively reflect potential relationships between risk factors by connecting probability distributions on a finite set of random variables. Directed edges represent statistical or causal dependencies between variables and the conditional probability distribution quantifies the effect of the parent on the child node (16).

The primary aim of this research is to develop a new predictive nomogram to guide clinicians in identifying patients at high risk of EJAL and intervening. Subsequently, we analyzed the relationships among the risk factors through the Bayesian network model. This integrated approach not only quantifies individual EJAL risk probability, but also reveals modifiable interaction pathways, thereby empowering personalized preoperative optimization and fundamentally advancing beyond conventional risk scoring systems.

## 2 Materials and methods

### 2.1 Patients

This research included 238 patients who performed total gastrectomy and esophagojejunal Roux-en-Y anastomosis for gastric cancer between January 2017 and June 2022 in the Department of Gastrointestinal Surgery of the First Hospital of Shanxi Medical University and was approved by the institutional review board of First Hospital of Shanxi Medical University (No. KK0191).

The inclusion criteria were as follows: (1) pathologically confirmed gastric cancer origin; (2) performed total gastrectomy; (3) complete preoperative data and clinicopathological characteristics. The exclusion criteria were as follows: (1) combination of other organ malignancies; (2) severe co-morbidity such as autoimmune disease, blood disease, obviously abnormal liver function or kidney function; (3) undergoing palliative resection surgery.

### 2.2 Data collection

We retrospectively collected 32 variables that may be associated with the occurrence of EJAL, comprised of patient-related variables [gender, age, body mass index (BMI), history of smoking and drinking, hypertension and diabetes mellitus (DM)], surgery-related variables [type of anastomosis (ToA), duration of operation, procedural approach, combined organ resection], tumor-related variables (tumor location, differentiation, pathological type, T stage, N stage, maximum tumor diameter, nerve invasion, vascular invasion, and neo-adjuvant chemotherapy), peripheral blood inflammatory and nutritional indexes 1 week before surgery [albumin (ALB), prealbumin (PA), white blood cell (WBC), hemoglobin (HB), globulin (GLO), neutrophil (Neut), lymphocyte (Lymph), blood platelet (PLT), neutrophil-to-lymphocyte ratio (NLR), lymphocyte-to-monocyte ratio (LMR), systemic inflammation response index (SIRI), albumin-to-globulin ratio (AGR)]. The TNM of gastric cancer was classified according to the American Joint Committee on Cancer (AJCC) Staging Handbook (8th edition).

### 2.3 Diagnosis of EJAL

Esophagojejunal anastomotic leakage is considered to be present when a patient has these features on several diagnostic tests as follows: (1)upper gastrointestinal contrast swallow showing contrast extravasate; (2)postoperative computed tomographic (CT) scan imaging suggests EJAL; (3)Presence of oral methylene blue in the peritoneal drainage fluid (17).

### 2.4 Definitions

The composite indicators in this research are defined as follows. NLR = neutrophils (10^9^/L)/lymphocytes (10^9^/L); SIRI = neutrophils (10^9^/L) × monocytes (10^9^/L)/lymphocytes (10^9^/L); AGR = albumin (g/L)/globulin (g/L); LMR = lymphocytes (10^9^/L)/monocytes (10^9^/L). Drinking history was defined as average of ≥ 1 drink per week for more than 6 months, or previously meeting this criterion and abstaining from alcohol for less than 6 months. Smoking history was defined as an average of ≥ 1 cigarette/day for more than 6 months before hospitalization. Several laboratory indicators are classified into lower, normal, and higher groups according to the hygiene criteria formulated by the National Health Commission of the People’s Republic of China. Hemoglobin (Hb) < 13 g/dL for males or Hb < 12 g/dL for females is identified as anemia according to World Health Organization (WHO) criteria.

### 2.5 Statistical analysis

Statistical analysis was performed with the SPSS (version 21.0) and R Studio (version 4.3.2) software programs. A *p* < 0.05 was considered as statistically significant.

According to the diagnostic criteria of EJAL, patients were categorized into the EJAL group and no EJAL group. Continuous variables were expressed as mean ± standard deviation (SD) or median, and categorical variables were expressed as frequencies and percentages. The optimal cut-off value of preoperative nutritional indexes, composite indexes, and operative duration was determined by the receiver operating characteristic (ROC) curve.

The chi-square test was used for univariable analysis to select potential predictors, which were then incorporated into multivariable logistic regression. The predictors were presented as odds ratio (OR) and 95% confidence interval (CI). A nomogram composed of these factors based on the logistic regression model was constructed. The receiver operating characteristic (ROC) curve and calibration curve of the nomogram model were developed to indicate the predictive ability of the model. In addition, the clinical benefit value of the nomogram model was assessed by decision curve analysis (DCA). Ultimately, a Bayesian network model was developed to analyze the interrelationship between the variables.

## 3 Results

### 3.1 Characteristics of the clinical baseline

The Clinical baseline characteristics of the patients are shown in Table 1. A total of 238 patients were included in this research. Patients were followed daily during hospitalization and at 30 days postoperatively. The median follow-up duration was 30 days (IQR: 28–35), covering the critical window for EJAL development. Among them, EJAL was observed in 13 patients (5.4%), with the median time to EJAL occurrence of 6 days (range: 4–10 days). The enrolled patients were 203 (85.3%) males and 35 (14.7%) females and the mean age was 65.43 ± 9.26 years.

**TABLE 1:** The baseline characteristics of the enrolled patients.

Variables	*n* (%)		χ^2^	*P*-value
	EJAL cases	No-EJAL cases		
Gender			0.110	0.740
Male	12 (92.3)	191 (84.9)	–	–
Female	1 (7.7)	34 (15.1)	–	–
Age (years)			1.433	0.231
< 60	1 (7.7)	60 (26.7)	–	–
≥ 60	12 (92.3)	165 (73.3)	–	–
Drinking history			4.367	0.037
Yes	8 (61.5)	67 (29.8)	–	–
No	5 (38.5)	158 (70.2)	–	–
Smoking history			0.626	0.429
Yes	7 (53.8)	96 (42.7)	–	–
No	6 (46.2)	129 (57.3)	–	–
Diabetes mellitus			8.504	0.004
Yes	5 (38.5)	20 (8.9)	–	–
No	8 (61.5)	205 (91.1)	–	–
Hypertension			0.712	0.399
Yes	5 (38.5)	54 (24.0)	–	–
No	8 (61.5)	171 (76.0)	–	–
BMI (kg/m^2^)			0.022	0.989
≥ 24	5 (38.5)	85 (37.8)	–	–
18.5–23.9	7 (53.8)	120 (53.3)	–	–
< 18.5	1 (7.7)	20 (8.9)	–	–
Type of anastomosis			4.927	0.026
End-to-side	9 (69.2)	86 (38.2)	–	–
Semi-end-to-end	4 (30.8)	139 (61.8)	–	–
Duration of operation (min)			0.179	0.672
≥ 259	5 (38.5)	65 (28.9)	–	–
< 259	8 (61.5)	160 (71.1)	–	–
Tumor location			0.420	0.811
Upper	9 (69.2)	138 (61.3)	–	–
Middle	2 (15.4)	51 (22.7)	–	–
Lower	2 (15.4)	36 (16.0)	–	–
pT			0.509	0.476
1∼2	4 (30.8)	42 (18.7)	–	–
3∼4	9 (69.2)	183 (81.3)	–	–
pN			0.398	0.528
0∼1	6 (46.2)	124 (55.1)	–	–
2∼3	7 (53.8)	101 (44.9)	–	–
Neurological violation			0.060	0.806
Yes	8 (61.5)	146 (64.9)	–	–
No	5 (38.5)	79 (35.1)	–	–
Vascular thrombosis			0.198	0.656
Yes	4 (30.8)	83 (36.9)	–	–
No	9 (69.2)	142 (63.1)	–	–
Differentiation extent			0.861	0.353
Low ∼ medium-low	8 (61.5)	165 (73.3)	–	–
Medium ∼ high	5 (38.5)	60 (26.7)	–	–
Pathological type			0.156	0.693
Adenocarcinoma	10 (76.9)	183 (81.3)	–	–
Non-adenocarcinoma	3 (23.1)	42 (18.7)	–	–
Maximum diameter of tumor (cm)			2.454	0.117
< 4	12 (92.3)	152 (67.6)	–	–
≥ 4	1 (7.7)	73 (32.4)	–	–
Neo-adjuvant chemotherapy			1.471	0.225
Yes	0 (0)	23 (10.2)	–	–
No	13 (100)	202 (89.8)	–	–
Procedural approach			0.068	0.795
Laparoscopy	11 (84.6)	196 (87.1)	–	–
Open	2 (15.4)	29 (12.9)	–	–
Combined organ resection			0.224	0.636
Yes	3 (23.1)	32 (14.2)	–	–
No	10 (76.9)	193 (85.8)	–	–
ALB (g/L)			7.501	0.006
< 33.6	6 (46.2)	31 (13.8)	–	–
≥ 33.6	7 (53.8)	194 (86.2)	–	–
WBC			2.214	0.331
Lower	0 (0)	20 (8.9)	–	–
Normal	13 (100)	192 (85.3)	–	–
Higher	0 (0)	13 (5.8)	–	–
Preoperative anemia			1.302	0.254
Yes	7 (53.8)	77 (34.2)	–	–
No	6 (46.2)	148 (65.8)	–	–
Lymph			1.283	0.527
Lower	3 (23.1)	37 (16.4)	–	–
Normal	9 (69.2)	181 (80.4)	–	–
Higher	1 (7.7)	7 (3.2)	–	–
PA			0.087	0.958
Lower	4 (30.8)	74 (32.9)	–	–
Normal	9 (69.2)	150 (66.7)	–	–
Higher	0 (0)	1 (0.4)	–	–
GLO			0.991	0.609
Lower	0 (0)	14 (6.2)	–	–
Normal	13 (100)	209 (92.9)	–	–
Higher	0 (0)	2 (0.9)	–	–
PLT			1.302	0.522
Lower	0 (0)	20 (8.9)	–	–
Normal	12 (92.3)	192 (85.3)	–	–
Higher	1 (7.7)	13 (5.8)	–	–
Neut			2.451	0.294
Lower	0 (0)	23 (10.2)	–	–
Normal	13 (100)	189 (84.0)	–	–
Higher	0 (0)	13 (5.8)	–	–
SIRI			6.066	0.014
< 1.18	7 (53.8)	190 (84.4)	–	–
≥ 1.18	6 (46.2)	35 (15.6)	–	–
LMR			5.502	0.019
< 2.63	8 (61.5)	61 (27.1)	–	–
≥ 2.63	5 (38.5)	164 (72.9)	–	–
NLR			4.445	0.035
< 2.37	4 (30.8)	144 (64.0)	–	–
≥ 2.37	9 (69.2)	81 (36.0)	–	–
AGR			1.385	0.239
< 1.42	7 (53.8)	76 (33.8)	–	–
≥ 1.42	6 (46.2)	149 (66.2)	–	–

Data are shown as *n* (%). BMI, body mass index; ALB, albumin; PA, prealbumin; TP, total protein; WBC, white blood cell; GLO, globulin; Neut, neutrophil; Lymph, lymphocyte; PLT, blood platelet; SIRI, systemic inflammation response index; LMR, lymphocyte-to-monocyte ratio; NLR, neutrophil-to-lymphocyte ratio; AGR, albumin-to-globulin ratio.

The results of the univariable analysis indicate that the differences in drinking history (*p* = 0.037), DM (*p* = 0.004), type of anastomosis (*p* = 0.026), ALB (*p* = 0.006), SIRI (*p* = 0.014), LMR (*p* = 0.019) and NLR (*p* = 0.035) were statistically significant.

### 3.2 Incidence and treatment of EJAL

In this research, EJAL was observed to occur in 13 of 238 (5.4%) patients. Of these 13 patients, 10 patients were successfully discharged with adequate drainage and anti-infective treatment, one patient improved after endoscopic treatment, one patient of EJAL secondary to systemic infection was admitted to the ICU, and only one patient died within 30 days due to EJAL.

### 3.3 Screening for independent risk factors of EJAL

**FIGURE 1:**
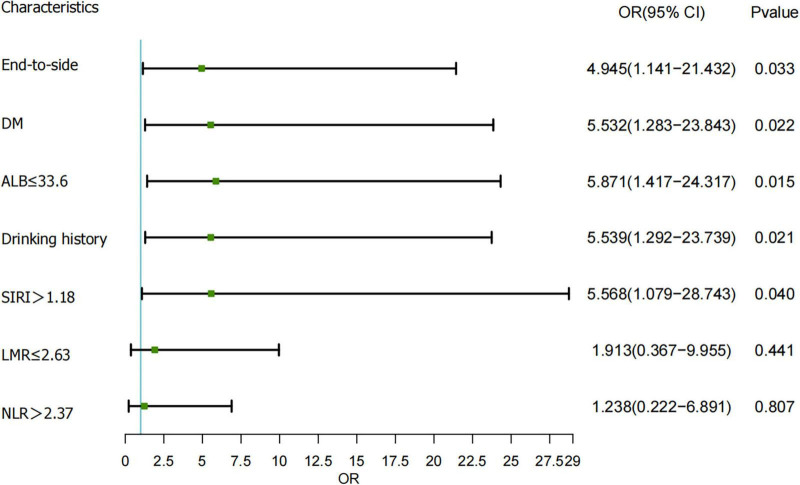
Multivariable logistic regression analysis to determine risk factors for esophagojejunal anastomotic leakage (EJAL). OR, odds ratio; CI, confidence interval; DM, diabetes mellitus; ALB, albumin; SIRI, systemic inflammation response index; LMR, lymphocyte-to-monocyte ratio; NLR, neutrophil-to-lymphocyte ratio.

The results of the multivariable logistic regression are shown in Figure 1, and anastomosis of the end-to-side [OR = 4.945, 95% CI (1.141−21.432)], DM [OR = 5.532, 95% CI (1.283−23.843)], ALB < 33.6 g/L [OR = 5.871, 95% CI (1.417−24.317)], Drinking history [OR = 5.539, 95% CI (1.292−23.739)] and SIRI ≥ 1.18 [OR = 5.568, 95% CI (1.079−28.743)] were identified as the ultimate independent risk factor.

### 3.4 Construction and evaluation of the nomogram

**FIGURE 2:**
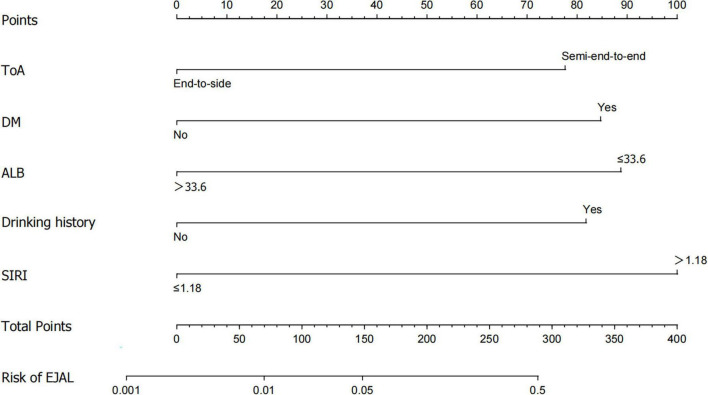
Nomogram for predicting esophagojejunal anastomotic leakage (EJAL) risk with predictive factors. ToA, type of Anastomosis; DM, diabetes mellitus; ALB, albumin; SIRI, systemic inflammation response index.

**FIGURE 3:**
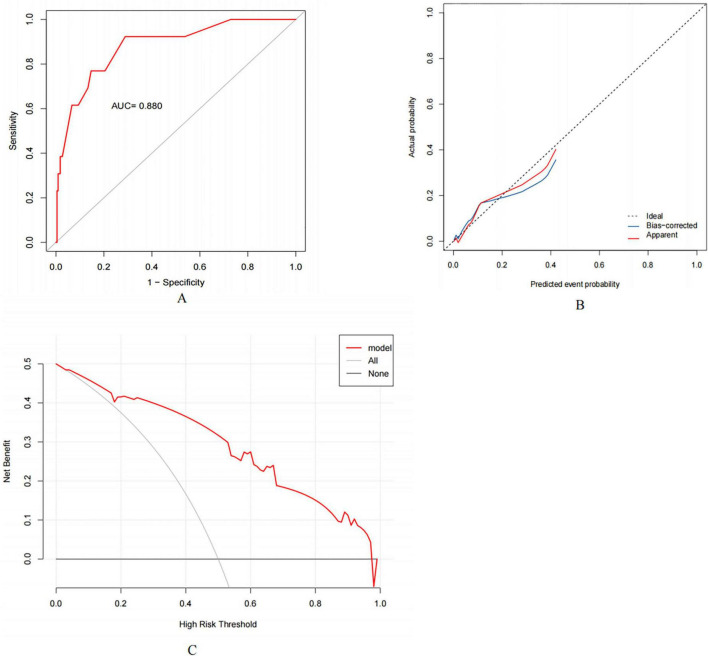
The evaluation of the nomogram. **(A)** The receiver operating characteristic (ROC) curve of the nomogram model for predicting esophagojejunal anastomotic leakage (EJAL). AUC is the area under the curve. **(B)** Calibration curves of the nomogram model. The x-axis represents the predicted EJAL probability and the y-axis represents the actual probability of EJAL. The diagonal black dashed line represents a perfect prediction by an ideal model. The red solid line represents the performance of the nomogram, and the blue solid line is bias-corrected by bootstrapping (B = 1,000 repetitions), indicating observed nomogram performance. **(C)** The decision curve analysis (DCA) of the nomogram model. The x-axis shows the threshold probability. The y-axis represents the net benefit. The thinner line represents the assumption that all patients have EJAL. The thicker line represents the assumption that no patients have EJAL.

A nomogram containing the aforementioned factors was constructed (Figure 2). The receiver operating characteristic (ROC) curve of the model was developed and the area under the curve (AUC) was 0.880 (95% CI: 0.782–0.978) (Figure 3A). The calibration curve for the nomogram indicating good agreements between the observed and predicted results (Figure 3B). Both indicate good predictive ability of the nomogram model. The decision curve analysis (DCA) revealed the model maintains higher net benefit at a wide threshold range (4%–97%) (Figure 3C).

### 3.5 The relationship between the variables based on the Bayesian network model

**FIGURE 4:**
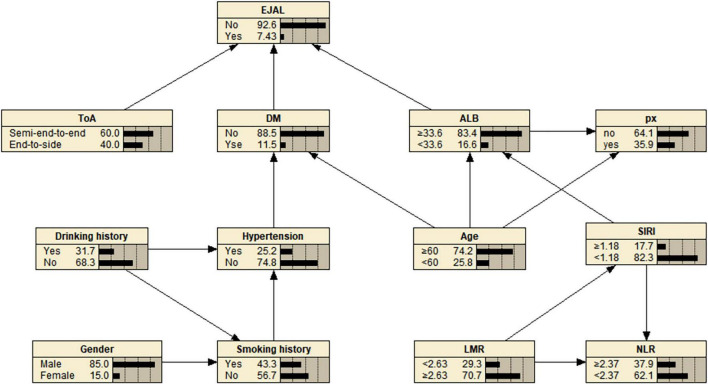
Bayesian network model of esophagojejunal anastomotic leakage (EJAL) risk factors. ToA, type of anastomosis; DM, diabetes mellitus; ALB, albumin; SIRI, systemic inflammation response index; LMR, lymphocyte-to-monocyte ratio; NLR, neutrophil-to-lymphocyte ratio.

A Bayesian network model (containing 13 nodes, 16 directed edges) of EJAL and its associated factors is further constructed (Figure 4). The results show that type of anastomosis (ToA), DM, and ALB was directly associated with the development of EJAL, while gender, age, drinking history, smoking history, hypertension, and SIRI were conditionally dependent on EJAL given the presence of mediator variables. Of these, hypertension and advanced age were directly associated with DM, while smoking history and drinking history were secondarily linked to DM through intermediary pathways. Also, there was a degree of dependence between ALB, advanced age, and higher SIRI.

The conditional probability distribution of EJAL with ToA, DM, and ALB as parent nodes indicates that the highest incidence of EJAL was 75% when the patient with DM and lower ALB underwent end-to-side anastomosis, and the incidence is the lowest (0.9%) when the conditions are reversed (Supplementary Table 1). Likewise, according to the conditional probability distribution of DM with age and hypertension as the parent node, DM is most common in hypertensive patients of advanced age (Supplementary Table 2).

## 4 Discussion

Esophagojejunal anastomotic leakage is a life-threatening complication following total gastrectomy. We developed a nomogram incorporating five risk factors to predict EJAL risk. The model demonstrated good predictive power and clinical application in the receiver operating characteristic (ROC) curve, calibration curve, and decision curve analysis (DCA). Bayesian network modeling further quantified risk factor interdependencies through conditional probability tables and directed edges, identifying actionable intervention pathways.

During surgical procedures for esophagojejunal Roux-en-Y anastomosis, the narrow operating space and the anatomical position of the esophagus make anastomosis of the esophagus and jejunum the most difficult step. However, the optimal procedure that also complies with the principles of tumor resection and effectively reduces the incidence of short- and long-term postoperative complications is controversial and has also been the focus of current debate and research. The current major mechanical anastomosis are circular and linear stapler methods, a recent meta-analysis found no statistically significant difference in EJAL incidence between these two techniques following total gastrectomy (18). The linear staplers are mainly used to perform esophagojejunal side-to-side anastomosis, such as overlap, π-shaped anastomosis, and functional end-to-end anastomosis (FEEA) (19–21).

It is undeniable that side-to-side anastomosis simplifies the operation of esophagojejunal anastomosis and creates a larger anastomosis. It has been reported that the probability of postoperative anastomotic stenosis is significantly lower in patients with side-to-side anastomosis anastomosis (22). However, the length of the free esophagus is more demanding when performing a side-to-side anastomosis using linear staplers. This requirement sometimes made it necessary to extend our operations toward the esophagus in the mediastinum, while ensuring the safety of the margin. Secondly, the anastomotic tension of side-to-side anastomosis is uneven, especially at the highest point of the anastomosis is the largest. While the circular staplers are mainly used to perform end-to-side anastomosis of the esophagojejunum, e.g., hand-sewn purse-string suture, single staple technique (SST), hemi-double staple technique (HDST), and OrVilTM (23–26). It is worth noting that these methods are all modifications of how the anvil can be more safely and easily placed in the esophagus. While Zhao et al. proposed a novel method called semi-end-to-end anastomosis that the circular stapler is inserted through a small incision of the jejunum (27). This method avoids obstruction of retraction when the anastomosis enters the esophageal hiatus. Thus, anastomotic tension and the incidence of EJAL are reduced. In the present research, we conclude that the semi-end-to-end anastomosis has a lower incidence of EJAL than the end-to-side anastomosis. It has been previously shown that semi-end-to-end anastomosis is effective in reducing the probability of anastomosis-related complications in patients after surgery, including anastomotic stenosis and leakage (28). In practice, anastomotic technique selection depends heavily on surgeon experience and preference. However, strict adherence to evidence-based indications is essential. Further research is warranted to establish standardized selection criteria.

In this research, we found that the preoperative albumin level may be the most critical factor contributing to the development of EJAL. According to the results of multifactorial logistic regression, patients with lower preoperative albumin levels were 5.871 times (95% CI: 1.417−24.317) more likely to develop an EJAL after surgery than those with higher preoperative albumin levels, which was the highest of the five risk factors. Albumin levels not only reflect the nutritional status of the body, but also play an important regulatory role in body fluid distribution, acid-base physiology, and substrate transport (29–31). A previous research has shown that low preoperative albumin levels lead to increased morbidity of postoperative complications and mortality rates (32). Likewise, preoperative low albumin has been identified as a risk factor for EJAL in previous researches (11, 33). We analyze the reason for this as poor nutritional status that does not provide the substances needed for anastomotic healing, which leads to poor tissue healing. In addition, low albumin levels mean that vascular colloid osmotic pressure is reduced, which will lead to increased inflammatory exudation around the anastomosis and severe tissue edema. The Systemic Inflammation Response Index (SIRI) is an indicator based on peripheral neutrophil, monocyte, and lymphocyte counts and reflects the status of the systemic immune response and inflammation (34). Several studies have pointed out the important predictive value of SIRI in the prognosis of various diseases, such as stroke, rheumatoid arthritis, renal, colorectal, and gastric cancer (35–40). Moreover, Schietroma et al. (41) found that patients with preoperative SIRI ≥ 0.82 were more likely to develop EJAL. The results of our research also show that a high level of preoperative SIRI is one of the risk factors for EJAL. This association may stem from inflammation-induced hypermetabolic consumption and heightened susceptibility to secondary infections. Preoperative optimization of nutritional status and inflammatory profiles is imperative through protocol-driven supplementation and anti-inflammatory regimens.

Diabetes mellitus is a group of systemic metabolic diseases, we found that DM [OR = 5.532, 95% CI (1.283−23.843)] is a risk factor for EJAL in the present research. DM has been reported not only to affect incision healing (42) but also to be detrimental to the growth of intestinal anastomosis (43). In the research by Maejima et al. (14), the risk of postoperative EJAL in diabetic patients was 5.18 times (95% CI: 1.77−15.13) higher than in non-diabetic patients. In another research, it was concluded that pre- and post-operative glycemic control was more important than the DM itself (44). Therefore, we need to be extra careful with diabetic patients undergoing esophagojejunal anastomosis, preoperative and postoperative glycemia should be controlled within an acceptable range as far as possible.

A history of alcohol consumption has been shown in some reports to increase the risk of anastomotic leakage after colorectal surgery (45–47). In this research, we found that a history of alcohol consumption increases the risk of EJAL. A systematic review of alcohol-related surgical risks indicates that chronic alcohol consumption induces hepatic injury, triggering coagulopathy and impaired nutrient synthesis. Concurrently, alcohol-induced immune dysregulation elevates susceptibility to postoperative infections (48). In addition, Wood S et al. (49) concluded in cellular experiments that chronic alcohol exposure renders intestinal epithelial cells vulnerable to bacterial infection, and alcohol-treated cells partially regain their ability to remedy the negative impact of alcohol after stopping ethanol treatment for two weeks. Therefore, it is crucial to stop alcohol intake in time before surgery.

Based on the results of the Bayesian network model it can be seen that patients with a history of smoking and alcohol consumption often have concomitant hypertension, whereas diabetes mellitus as a risk factor for EJAL is more common in hypertensive patients of advanced age than in patients with the opposite condition. The effects of smoking and alcohol consumption on the cardiovascular system are well-recognized that will lead directly to the development of hypertension. And hypertension and diabetes have similar risk factors, which makes hypertension and diabetes usually occur together (50). In a Chinese research, it was concluded that hypertensive patients had an 11.0% increased risk of developing diabetes (51). Additionally, lower albumin levels are more likely to be seen in patients with advanced age and higher SIRI levels. We consider that high levels of SIRI indicate an increased systemic inflammatory response in the patient, which would lead to increased albumin consumption. In the Bayesian network model, advanced age also seems to be a noteworthy factor. According to the results of Bayesian network model, lower albumin levels and diabetes were more common in patients with higher age than in those with lower age. This is coupled with the fact that older patients are less able to self-heal and tolerate surgery, and tend to have more comorbidities. Notably, cardiovascular risks such as hypertension escalate with age due to progressive vascular stiffening (52). Therefore, age remains a critical consideration in perioperative risk assessment.

As a single-center retrospective study, our analysis was restricted to semi-end-to-end versus end-to-side anastomosis. The modest cohort size also limits generalizability. Future work will expand sample sizes, conduct prospective validation, and implement multicenter verification.

## 5 Conclusion

Surgeons should be alert to the occurrence of EJAL, especially in patients with end-to-side anastomosis, DM, drinking history preoperative lower ALB, and higher SIRI. Also, males, advanced age, smoking history, and hypertension can affect the development of EJAL.

## Data Availability

The raw data supporting the conclusions of this article will be made available by the authors, without undue reservation.
