# Supplementary material for: Analysis of risk factors for esophagojejunal anastomotic leakage after total gastrectomy based on Bayesian network model
Source: Front Med (Lausanne). 2025 Aug 5;12:1632214. doi: 10.3389/fmed.2025.1632214 (PMC12361184; doi:10.3389/fmed.2025.1632214)
Supplement: Supplementary file 1 [file Table_1.DOCX]

**Table S1** Conditional probability distribution of EJAL with ToA, DM, and ALB as parent node

| Parent node | | | EJAL(%) | |
| --- | --- | --- | --- | --- |
| Type of Anastomosis | DM | Lower ALB | Yes | No |
| End-to-side | yes | yes | 75.0 | 25.0 |
| End-to-side | yes | no | 33.3 | 66.7 |
| End-to-side | no | yes | 8.3 | 91.7 |
| End-to-side | no | no | 7.7 | 92.3 |
| Semi-end-to-end | yes | yes | 28.6 | 71.4 |
| Semi-end-to-end | yes | no | 7.7 | 92.3 |
| Semi-end-to-end | no | yes | 18.2 | 81.8 |
| Semi-end-to-end | no | no | 0.9 | 99.1 |

ToA, type of anastomosis; DM, diabetes mellitus; ALB, albumin.

**Table S2** Conditional probability distribution of DM with age and hypertension as parent node

| Parent node | | DM(%) | |
| --- | --- | --- | --- |
| Age | Hypertension | Yes | No |
| ≥60 | Yes | 26.4 | 73.6 |
| ≥60 | No | 9.4 | 90.6 |
| <60 | Yes | 10.0 | 90.0 |
| <60 | No | 3.6 | 96.4 |

DM, diabetes mellitus.
